# Vision-Based Approach in Contact Modelling between the Footpad of the Lander and the Analogue Representing Surface of Phobos

**DOI:** 10.3390/s21217009

**Published:** 2021-10-22

**Authors:** Marek Cała, Piotr Kohut, Krzysztof Holak, Daniel Wałach

**Affiliations:** 1 Department of Geomechanics, Civil Engineering and Geotechnics, Faculty of Civil Engineering and Resource Management, AGH University of Science and Technology, al. Mickiewicza 30, 30-059 Kraków, Poland; cala@agh.edu.pl; 2 Department of Robotics and Mechatronics, Faculty of Mechanical Engineering and Robotics, AGH University of Science and Technology, al. Mickiewicza 30, 30-059 Kraków, Poland; pko@agh.edu.pl (P.K.); holak@agh.edu.pl (K.H.)

**Keywords:** vision systems, contact modelling, Phobos analogue

## Abstract

Identifying solar system surface properties of celestial bodies requires the conducting of many tests and experiments in conditions similar to those found on various objects. One of the first tasks to be solved by engineers is determining the contact condition between the lander and the surface of a given celestial body during landing in a microgravity environment. This paper presents the results of experimental studies and numerical simulations of the contact phenomenon between the lander foot model and the Phobos analogue. The main goal of the experimental tests was to obtain measured deformation data of the studied analogues using 2D and 3D vision systems, which were employed to analyze the behavior of the lander foot and the surface of the studied analogue itself and to calibrate the numerical models. The analogue representing the Phobos surface was foam concrete. The variable parameters in the study were the analogue thickness and the lander foot velocity at the time of contact. Tests were conducted for three different contact velocities of 1.2 m/s, 3.0 m/s, and 3.5 m/s. Taking into account the mass of the lander foot model, kinetic energies of 30.28 J, 189.22 J, and 257.56 J were obtained. The results showed that at low contact velocities, and thus low kinetic energies, no significant differences in behavior of the material directly under the lander foot were observed, and similar values of forces in the lander foot were obtained. For higher contact velocities, the behavior of analogues with varying thicknesses was different, resulting in different values of analogue deformation and dynamics of increments and decrements of force in the lander foot itself. Although performed on a single material, the experiments revealed different behaviors depending on its thickness at the same impact energy. This is an essential guideline for engineers who need to take this fact into account when designing the lander itself.

## 1. Introduction

In recent years, advanced image processing methods and computer vision systems have found applications in many branches of science and industry. Non-contact measurement has become one of the most important techniques to obtain information on static states and dynamic processes of the engineering structures. The basic principle of vision-based methods is an analysis of information encoded in the light reflected from surfaces of objects and captured in the form of 2D images by a camera’s sensor, or in the form of video files, which additionally contain temporal changes of the observed object’s image. This approach makes it suitable for a measurement in which a direct contact of a sensor with the measured structure is undesirable or impossible due to geometrical limitations.

In the mechanical and civil engineering applications of computer vision, there are several important groups of solutions, which have been used in lab studies as well as in industrial and commercial systems. The first group includes surface state assessment methods.

The first methods belonging to this group were heuristic algorithms [1]. Researchers developed methods for the detection of damage types, such as cracks, delamination, and spalling for concrete structures, and fatigue cracks and corrosion for steel parts [2]. However, these approaches proved to be difficult for practical use, due to the high variation of pixel intensity values corresponding to features other than damage, such as shadows, moisture, and vegetation found in real structures. The introduction of artificial intelligence (AI) in general, and deep learning (DL) with convolutional neural networks (CNN) in particular [3,4], expanded the capability and robustness compared to the classical vision methods. Three AI approaches are presented in the literature: image classification, object or region detection, and semantic segmentation. In the case of the first approach, the entire image of the surface is classified as healthy or containing damage. Further processing may be done to classify damage into groups of increasing severity. In object or region detection, groups of pixels representing damage are detected and marked on the image by rectangular regions. The most advanced is the method of semantic segmentation in which each pixel is classified as belonging to a healthy part of the surface or damage of different kinds [5]. Ai et al. [6] developed a vision-based method for automatic crack growth monitoring based on video data. It was specifically designed for an analysis of rocks and rock-like materials. In this approach, CNN was applied for crack detection with improved accuracy due to the introduction of a Bayesian reference mechanism. Temporal and spatial correlations in video data were included in the analysis. An example of a practical system for crack detection was presented in [7]. The monitoring system was designed for tunnel inspection using cameras. Damage such as cracks of concrete walls was recognized using object-based classification and optical flow analysis.

A lot of work has been done to increase the automation level of image-based structural damage detection. An interesting application is automatic structural element recognition, which is done before an assessment of the elements’ states [8]. The transfer learning method is applied to classify between damage and undamaged members. CNN, which has already been pre-trained to recognize structural components, is used [9]. The method uses automatic bridge component recognition with the DL approach to recognize structural components of bridges [10] and also to recognize bridge structure as a whole within a background, distinguishing between the bridge and non-bridge objects.

The second group includes solutions for measurement and monitoring of structural displacements of objects under static and dynamic loads [11]. There are systems which observe a single point or several chosen crucial points using a distant camera equipped with a lens with high focal length. Recently, there have been reported many examples of systems designed for the full-field displacement or deformation course computation [12]. Multi-camera systems provide a series of images which may be processed to obtain 3D structure and deformation. The most common type of systems are stereovision systems that use a pair of cameras. For example, the vision system presented in [13] was used to compute a full-field deformation field of concrete filled steel tubular columns. Sun et al. [14], applied a calibrated stereo-camera system and CNN to measure asphalt mixture deformation to determine its modulus of elasticity and Poisson ratio. Tracked markers were in the form of black dots on a white background. Strain measurement of a reinforced concrete (RC) wall was presented in [15]. A two-camera system was used as well, but the surface of the wall was covered with a speckle pattern. Stereo-matching and tracking was carried out by the digital image correlation (DIC) method. On the other hand, the paper [16] reports an application of a four-camera system for deformation measurement. The analyzed object was an automobile tire subjected to loads. Four cameras were calibrated and synchronized. The automatic matching algorithm was applied based on simple black and white markers, each containing special visual code for recognition. A multi-camera system for the full-field measurement of deformation and strain fields of recycled aggregate concrete-filled steel tubular columns was presented in [17]. In the method, the corresponding points were tracked by dynamic surface tracking, and the full 3D deformation surface was reconstructed by a multi-ocular vision coordinate association. Additionally, the measurement of the system’s accuracy of reconstruction was improved further, as presented in [18], by the introduction of an adaptive point cloud correction algorithm. The introduction of sub-pixel methods allowed the resultant deflection curves to be smoother and more accurate [12]. In all solutions, tracked points may be special markers placed on the objects or natural features if the contrast is high enough. The deformation is often computed by the DIC method. In such cases, a high-contrast image noise pattern is placed on the structure before the measurement session. Video data contain additional temporal information which may be applied to find changes in the structure or compute its dynamic response. Introduction of high speed cameras made it possible to analyze vibration-related phenomena, such as the computation of frequency response function or performing modal analysis [19,20,21].

In the case of static deflection measurement, many vision-systems have been reported in the literature. One of the earliest practical examples is work by Yoneyama [22]. The single camera DIC method and additional movement correction was applied to measure the deformation field of the bridge under a truck load. The method was tested in various lightning conditions. Vision-based systems have been used for computation of structure deformation under load in industrial and civil engineering scenarios. For example, in the former case, a crane lifting weight was monitored by a pair of cameras [23]. In the latter case, a camera system was applied for monitoring of a tram viaduct deflecting under passing vehicle loads [12]. Many more applications of vision-systems for large scale structures deformation analysis have been reported [24]. Jeon et al. [25] described a many-camera system consisting of pairs of cameras, laser projectors, and projector screens for monitoring the entire span of the long-span bridge. In a paper by Poozesh et al. [26], an integrated multi-camera DIC system was applied for photogrammetric testing of wind turbine blades. Each camera pair provided a 3D reconstruction of the blade, and the entire 3D model was generated due to known calibration between all cameras in the vision system. An introduction of homography mapping made it possible to capture the structure’s images by a camera placed at an arbitrary point in space [27].

Feng et al. [28,29] presented a single camera system designed to track special optical markers as well as natural features like bolts and nuts. The method was tested on the railway bridge. The proposed approach was augmented to obtain the dynamic response of the observed structure as well. Many systems capable of dynamic measurements have been reported [30]. For example, Greenbaum [31] showed an application of a single camera for the computation of 3D rocking motion of structural and non-structural elements. Similarly, a 3D structure and motion reconstruction system using a single camera was presented in [32]. A model of a planar specimen attached to the surface of the analyzed object and homography transformation was applied. The developed method was tested in field experiments on a pedestrian footbridge deflected under an imposed load. A parallel, binocular vision system was applied by Xu et al. [33] for a real-time monitoring of rock dynamic deformation and disastrous failure prevention in deep underground coal mines. Displacement of underground roadway walls was measured, and a multi-index warning criterion was developed based on obtained data. The vision-based system can be augmented by additional scanning devices. In [34], a two-camera vision measurement system supported with depth sensing capabilities (RGB-D camera) was used to measure full-field dynamic displacements of structures.

Dynamic vision-based systems have been applied in the fatigue testing for an analysis of crack growth. For example, Carroll et al. [35] used high resolution DIC measurement for strain accumulation during the fatigue crack growth in polycrystalline Ni alloys. Mahal et al. [36] applied the same measurement technique to observe fatigue behavior of RC concrete beams under cyclic load conditions. A single camera was used to obtain strain fields in x and y dimensions. Pieczonka et al. [37] employed a high-speed camera and DIC for a damage detection method based on nonlinear crack–wave interactions in an aluminum beam with a fatigue crack. Quantitative analysis of the crack opening–closing action was carried out to exploit the process of nonlinear wave modulations. High speed cameras and the DIC method are often used in the analysis of mechanical properties of biological specimens, which undergo large deformations when subjected to a load. For example, the paper [38] presents a vision-based analysis of human Achilles’ tendon properties tested in the uniaxial tension test.

Based on previous applications of vision systems, an interesting direction for the use of these modern tools turned out to be the investigation of the lander foot contact with the surface analogue of celestial bodies. The prediction of material behavior in micro or zero-gravity conditions has not been sufficiently recognized yet. Predicting the properties of materials, liquids, mixed systems, or their behavior during contact with another object (e.g., lander) is crucial in determining the success of projects related to the exploration of celestial bodies (planets, the Moon, moons of other planets). Therefore, the research program was prepared, the aim of which was to describe the phenomenon of lander foot contact with one of the moons of Mars, Phobos, based on experimental studies and numerical simulations. The project was a response to a request of the European Space Agency to place landers on the surface of celestial bodies—Phobos, Deimos, and on our own natural satellite, the Moon. This is perhaps not particularly surprising, as landing missions undertaken in the past have been extremely successful. Projects such as ESA Cassini-Huygens (landing on Titan) and RosettaPhilae (first landing on a comet surface) and JAXA Hayabusa (landing on a near-Earth asteroid) have provided important scientific information about the structure and properties of small celestial bodies. The significance of space exploration should be considered, in light of the Global Exploration Roadmap [37] announced by ESA and ISECG, as a crucial aspect of international cooperation activities based on a common set of exploration objectives and identified benefits for humanity. It is believed that the first stage of missions related to the global strategy is going to focus on autonomous spacecraft. Efficient landing algorithms and appropriate mechanical designs for landers will be essential to the successful future endeavors. From the range of past, ongoing, and planned landing missions, it is clear that the landing process itself is a key to the success of the entire project. There is no doubt that missions such as Phootprint would benefit greatly from the results of the subject project, which was named LOOP (Landing Once On Phobos). The name originated from the fact that, while working on the REST project (Robotically Enhanced Surface Touchdown, Phootprint mission), a team involved in the project realized that an empirically validated contact model would provide a valuable tool for various projects involving the landing process. The model should be simultaneously developed with its corresponding laboratory model. The scope of the whole project in its assumptions was directly related to Phobos, but the results could serve the general purpose of landing on other celestial bodies. The developed contact model will be used to create a simplified engineering model that will be easy to implement in lander control systems.

Taking into account the objectives of the project, it became necessary to perform experimental tests, the main task of which was to obtain measured data of deformation of tested analogues during their contact with the lander footprint model. This paper presents the methodology and results of such tests, which will provide a basis for the design of appropriate shock-absorbing systems for future landers. The developed experimental program and test stand represent a novel approach to the analyzed technical problem. This results from the necessity of combining various scientific areas such as mechanics, measurement systems, geotechnics, and numerical simulations. Previous research work covered mainly selected aspects of the raised problem, such as the description of the properties of the analogue itself [39,40,41,42,43]. Due to the research problem, it seems that only an interdisciplinary approach to the subject may provide satisfactory results in terms of describing the phenomenon of contact between the lander’s foot and the surface of various celestial bodies.

## 2. Materials and Methods

### 2.1. Tested Analogue

The present knowledge of Phobos’ surface is based on high-resolution images and infrared mapping [44,45,46,47,48]. The origin of Phobos, and the mineral composition of its surface and bedrock require further study [49,50,51]. These factors are related to the geotechnical parameters of the surface. However, the geotechnical parameters can be estimated from the experience of previous missions to asteroids, the Moon, and Mars, as well as materials (analogues) intended to represent the surface of Earth and also from materials (analogues) intended to represent the surface of Phobos [39,40,41,42,43]. It should be noted that the variability of geotechnical parameters of different materials under limited gravity requires deeper investigation [52,53]. The surface behavior in response to a lander foot is difficult to predict. Therefore, a terrestrial material—foam concrete—was selected for testing, as an analogue of Phobos, with a highly porous structure that allows proper testing at the same time. Foam concrete is an ultra-light concrete (often with a density below 800 kg/m^3^), which was defined back in the 1950s in the PN-B 06565:1959 [54] standard as a lightweight artificial porous stone, with a mostly closed pore structure, formed from a foam concrete mass as a result of the completion of the cement setting process. The foam concrete is a mixture of cement, water, sand, and foaming agent. The exact composition of the foam concrete used was protected by the manufacturer.

**Figure 1:**
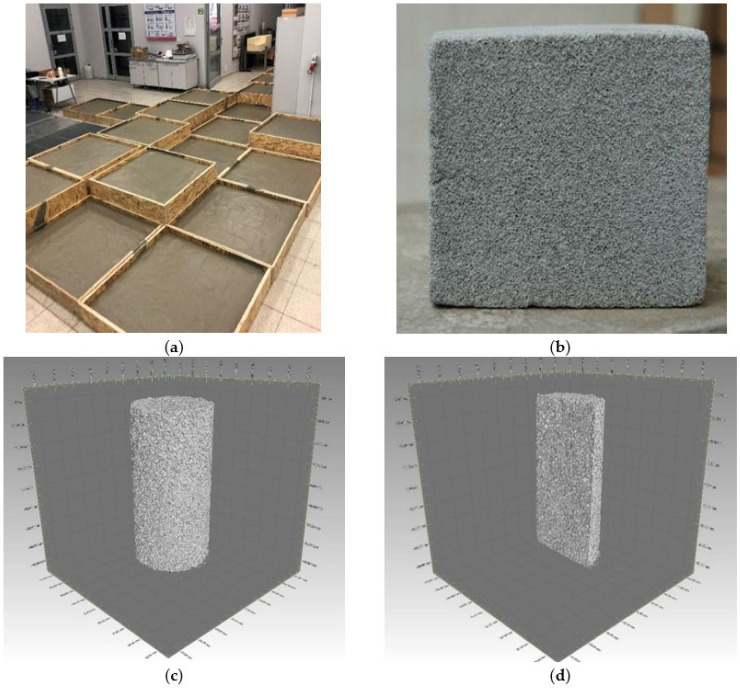
Research analogue made of foam concrete: (**a**) fabrication process of foam concrete slabs; (**b**) 15 × 15 × 15 cm cube sample extracted from slabs; (**c**) structural analysis using CT of ∅40/80 mm core sample; (**d**) vertical section of ∅40/80 mm core sample.

The test analogues were made as foam concrete slabs with dimensions of 100 × 100 cm and two thicknesses of 10 and 30 cm (Figure 1a). After 28 days, core samples ∅40/80 mm and cube samples 150 × 150 × 150 mm (Figure 1b) were extracted from the slabs, on which the basic physical–mechanical parameters were determined:

- volume density based on EN ISO 17892−2 [55],
- uniaxial compressive strength according to EN 12390−3:2009 [56],
- tensile strength using the Brazilian method based on EN 12390-6:2009 [57],
- Young’s modulus based on EN 12390−13:2014 [58]—method A,
- structural analysis using the computed tomography (CT) technique (Figure 1c,d).

It was assumed that in the lower layers of the Phobos surface, there is rigid material, so the base of the analogue model was made of concrete slabs of the C35/45 class. The total thickness of the test material during the tests was 1.0 m in the configurations of 70 cm of concrete and 30 cm of foam concrete analogue, and 90 cm of concrete and 10 cm of foam concrete analogue. For the concrete, similarly to the foam concrete, the basic physical and mechanical parameters were determined.

### 2.2. Testbed

**Figure 2:**
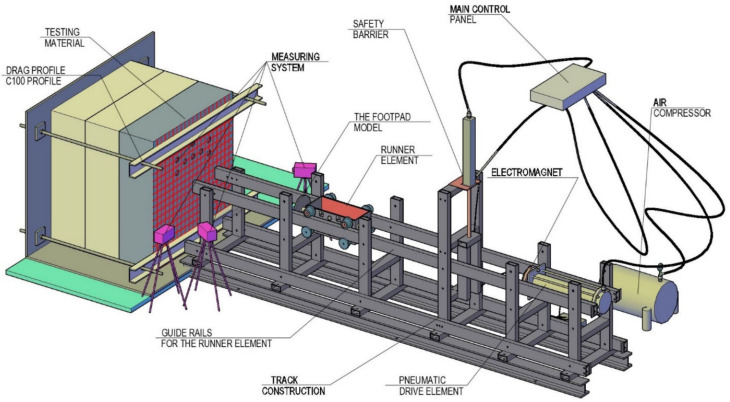
The LBT construction model.

The linear bearing testbed (LBT) enables testing of the contact of the lander foot with the Phobos analogue. The LBT was designed and constructed by the authors [59]. In order to minimize the influence of gravity on the reaction of the tested analogue (change of the direction of gravity), the stand was designed for testing at the horizontal level. The stand is equipped with a driving system that allows the contact to be tested at different velocities, which, with an adjustable runner element weight and lander footpad, enables different impact energies to be obtained. The test stand model is shown in Figure 2.

The test stand consists of the following subsystems:

- Drive system (pneumatic system of elements that trigger the movement of the runner element with the model of the footpad at the predefined velocity. This system consists of an air compressor and a pneumatic drive element);
- Runner element (the device that represents the weight of the lander, to which the footpad is attached);
- Electromagnet (the device that releases the runner element);
- Track structure (steel construction for linear movement of the runner element with the footpad model with minor sliding friction);
- Test analogue mounting system (a system of steel members enabling stable attachment of test analogues with different test surface inclination angles to the lander footpad);
- Measuring system;
- Safety system (the safety system is composed of a bi-directional pneumatic actuator powered by compressed air and a steel structure designed to block the pneumatic drive element from uncontrolled release);
- Main control panel (a panel that controls operation of the safety system, pneumatic drive element, and measuring system. The device is situated above the testbed localization).

**Figure 3:**
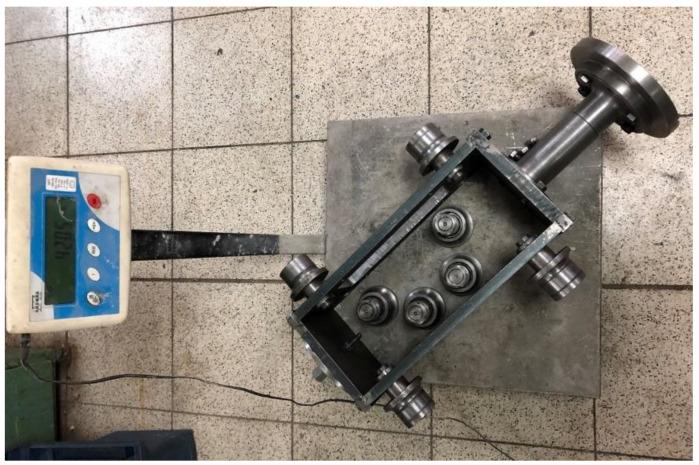
The runner element with attached footpad model.

The total mass of the runner element was 42.05 kg, and the lander foot diameter was 190 mm (Figure 3). Testing of individual analogue thicknesses was carried out for 3 velocities: 1.2 m/s, 3.0 m/s, and 3.5 m/s.

Given the total mass of the Runner Element and assumed velocities at the time of contact, experiments were conducted for lander foot kinetic energies of 30.28 J, 189.22 J, and 257.56 J.

### 2.3. Measuring System

**Figure 4:**
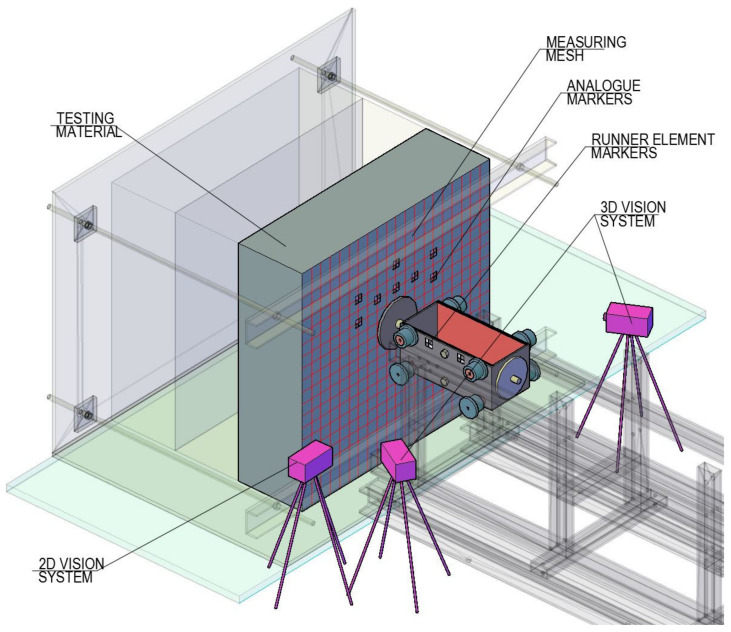
Schematic view of the localization of vision-based measurement systems in relation to the rest of the testbed elements.

**Figure 5:**
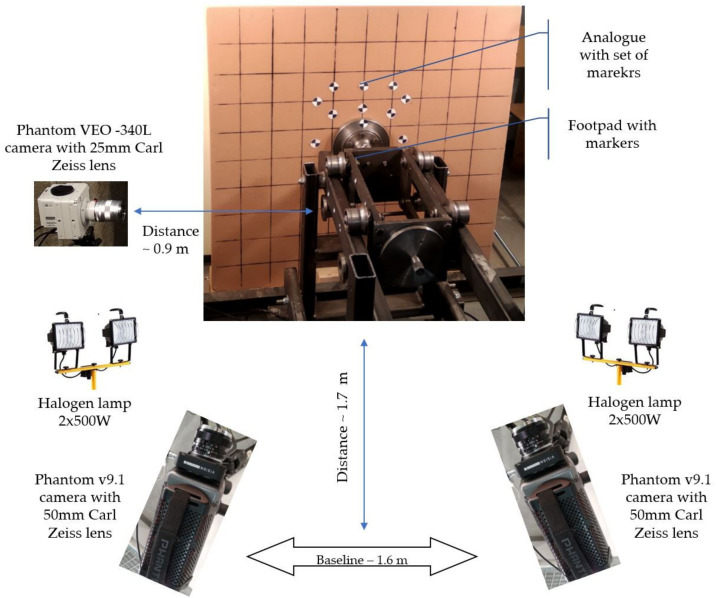
Detailed scheme of the 3D and 2D vision-based measurement system with additional equipment.

The developed vision-based measurement system was composed of a set of three high-speed cameras, an illumination system, and Tema-Automotive software [60]. Two high-speed Phantom v9.1 cameras (1632 × 1200 pixels) equipped with 50 mm focal length Carl Zeiss lenses constituted a 3D vision system, while the high-speed Phantom VEO−340L camera (2560 × 1600 pixels) with 25 mm focal length Carl Zeiss lens represented a 2D vision system (Figure 4 and Figure 5). As the illumination system, two halogen lamps 2 × 500 W were applied. For the image processing stage, Tema-Automotive software with quad tracker methods was proposed. Internal and external calibration procedures were provided utilizing a flat calibration plate. External camera calibration was carried out employing a relative camera orientation procedure implemented in TEMA software [60]. In the case of the 2D vision system, only internal calibration was performed to remove lens distortion. Tema-Automotive software was applied to calculate the velocity of the footpad and displacements of the analogue at the selected points. To detect and track markers, a quad tracker algorithm was chosen [60].

**Figure 6:**
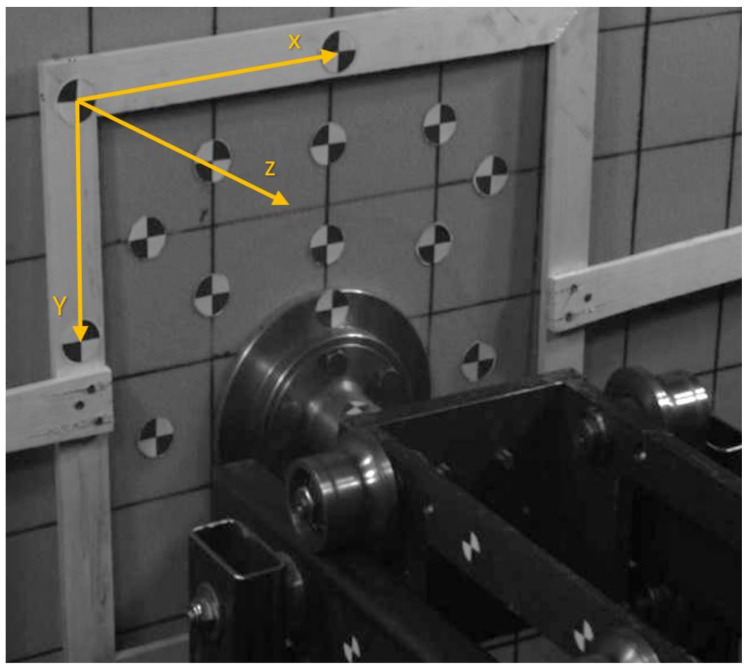
The arrangement of markers on the analogue surface.

A set of ten markers (Figure 5) was stuck on the analogue to calculate the 3D displacement in selected points, and two markers were placed on the footpad to calculate its velocity. The displacements of the analogue were calculated by the 3D vision system and the velocity of the footpad by employing the 2D vision system. In both cases, the quad tracker algorithm was applied to detect and track the markers. The location of the markers on the analogue was dictated by the limited field of view of the 3D camera system and by the construction of the test stand. Additionally, a wooden frame was prepared and placed parallel to the plane of the analogue on which three markers were attached. The frame was assumed to be motionless during the measurements. Thanks to the fixed reference points (markers on the wooden frame), the coordinates of the remaining markers could be defined in a coordinate system unambiguously related to the plane of the analogue. For this purpose, based on available procedures in Tema [60], a new coordinate system was defined, which was developed using these three markers (points) on the wooden frame (Figure 6) and the transformation of three-dimensional coordinates defined in the configuration of one of the cameras, to the coordinate system associated with the tested analogue.

Specifications of the 2D and 3D vision systems are presented in Table 1. The frame rate for both vision systems was adjusted to 1000 fps, and all cameras were synchronized by an external trigger.

#### 2.3.1. Three-Dimensional Vision System-Level of Measurement Noise

For the purpose of the 3D vision system measurement noise estimation, standard deviations of markers centers’ positions were computed for 100 image sequences. The mean value of the level of measurement noise for the 3D vision system equaled 0.015 mm (in image plane 0.010 mm, in-depth 0.024 mm). The resulting 3D calibration error represented by RMS parallax error for 1685.509 mm baseline amounted to 0.019 mm.

#### 2.3.2. Two-Dimensional Vision System-Level of Measurement Noise

To evaluate the measurement noise of a 2D vision system, standard deviations of marker center positions was computed based on 100 frames of the reference image sequence (no motion of runner element). Position measurement error amounted to a value of 0.0073 mm (0.0079 mm in the *x*-axis and 0.0065 mm in the *y*-axis).

### 2.4. Mapping the Analogue Deformation

As a result of the tests, a set of three-dimensional x, y, z coordinates of individual markers located on the surface of the analogues and two-dimensional x, y coordinates of markers located on the runner element were obtained. All measurements were obtained in the local coordinate systems of the 3D camera system and the 2D camera. Using the fixed markers mounted on the wooden frame, the x, y, z displacements of the remaining markers (measurement points) were obtained and transformed (as described in Section 2.3) to the coordinate system defined on the plane coinciding with the plane of the analogue. Due to the perpendicular position of the optical axis of the 2D camera with respect to the direction of movement of the runner element, the obtained x, y coordinates of the markers mounted on the runner element were scaled in SI units (mm). In order to compare the individual test results, the acquired data on the behavior of the runner element were brought to a single time point, i.e., the beginning of contact of the lander’s foot during the motion with the tested analogue, taking this moment of time as the zero point. Therefore, the locations of the runner element before contact with the analogue were presented with negative time values. The start of the contact of the lander’s foot with the analogue was determined from a stationary reference measurement of the location of the lander’s foot when it was entirely in contact with the analogue.

Due to the symmetry of the system, the symmetrical contact behavior of the analogue was also assumed for further analysis. Therefore, additional measurement points were introduced on the deformation maps of the analogue surfaces, being the mirror image of the existing markers with respect to the horizontal axis passing through the center of the lander foot. Additionally, virtual markers located near the outer edges of the analogue were introduced, assuming that their x, y, z coordinates remained unchanged during the examination. The deformation of the surface of analogues directly under the foot of the lander was assumed to be constant with values corresponding to the displacement of the foot itself. Hence, virtual markers were also introduced on the deformation maps of the analogue surfaces, the distribution of which did not extend beyond the area of the lander foot.

**Figure 7:**
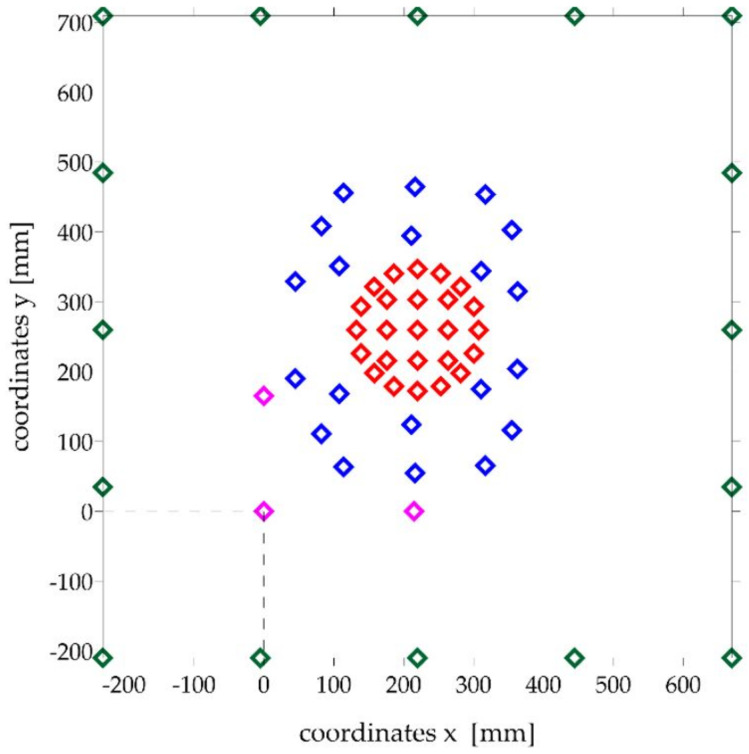
Location of measurement points used in the surface deformation analysis of analogues: blue markers located on the analogue surface and their mirror image; red markers (apparent) correspond to the displacement of the lander foot; purple markers define a coordinate system parallel to the surface of the analogue; green markers (apparent) are located on the edge of the analogue.

The origin of the coordinate system of all markers was brought to the coordinates of the fixed marker located on the wooden frame (Figure 7).

Using x, y, z coordinates of markers placed on the analogue and virtual markers, numerical deformation maps of the material surface from the moment of contact with the lander foot with a defined velocity were created. Numerical mapping based on an interpolation grid provides a measurable assessment of the efficiency of individual interpolation methods for various contact phenomena. Such analysis is particularly valuable in mapping areas with non-homogeneous data distribution. The maps were constructed in the Surfer program, which offers several interpolation methods: kriging, minimum curvature, nearest neighbor, natural neighbor, modified Shepard’s method, radial basis function, polynomial regression, inverse distance to a power, and triangulation with linear interpolation. However, as reported in the literature [61], only some of them are theoretically suitable for interpolation based on irregularly distributed data. These methods are kriging, minimum curvature, and polynomial regression.

**Figure 8:**
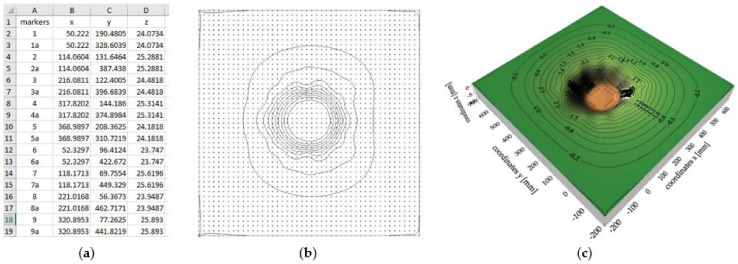
Process of generating numerical analogue surface deformation maps: (**a**) x, y, z data of individual markers; (**b**) interpolation grid; (**c**) a surface map and 3D deformation map of the analogue.

**Figure 9:**
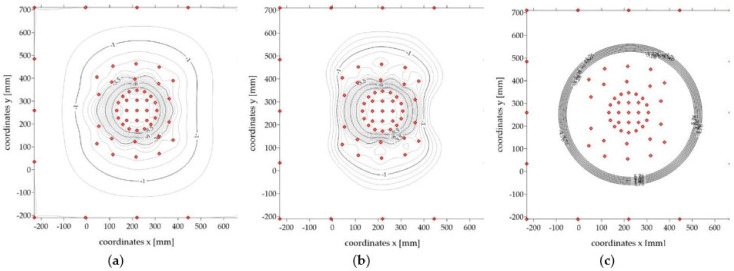
Surface deformation maps of the sample analogue created by selected interpolation methods: (**a**) kriging method; (**b**) minimum curvature method; (**c**) polynomial regression method.

The interpolation process results in the creation of an interpolation grid, which is the background used by the Surfer software to plot isolines (Figure 8). The interpolation process creates a very dense grid in spite of the irregularly distributed measurement points, which provides an accurate representation of the analyzed surface. Interpolation methods use a number of diverse algorithms to determine the magnitude of the interpolated parameters at nodal points. Each method uses a different algorithm to calculate the values at the nodal points and thus creates a different representation of the plotted surface. The choice of the interpolation method in the case of irregularly distributed measurement data, such as here, was dictated by the characteristics of the data set: the level of data distribution homogeneity, the number of points per unit area, the level of data variation, and the type of surface that the data reflect. The observation of the recordings from the tests, which verified the obtained deformation maps with the image registered during the experiment, was crucial in the analyzed case. Data interpolation was performed for the previously mentioned kriging, minimum curvature, and polynomial regression methods. The results of selected methods are presented in Figure 9.

Analyzing the obtained analogue surface deformation maps and comparing them to the deformations observed during testing of the tested materials, it was found that the kriging method gave the most reliable and realistic maps in the process of interpolation of non-homogeneous data, reflecting the course of the surfaces of the tested analogues; thus, this method was used for further analyses.

### 2.5. Numerical Simulations

Numerical simulations were performed using FLAC 7.0 3D software based on the finite difference method (FDM). This is one of the methods for the approximate solution of continuous boundary-initial problems. The essence of the FDM is to replace the derivatives with appropriate differential quotients defined over a discrete set of points. The method was initially applied to differential equations (local formulation) and was later extended to problems formulated in variational form (global formulation). In the standard formulation of the FDM, a regular grid of nodes is used. In the advanced formulation, an arbitrary mesh is used.

Using the FDM, a system of differential equations is solved to obtain the values of the field variables (i.e., stresses or strains) at the discrete points. One of the most widely used FDM programs in geotechnics is Itasca’s FLAC 3D software. It uses an explicit method of successive steps to solve a system of differential equations, after which it changes the system of equations each time. This enables the calculation of stress and deformation fields in the analyzed area, taking into account complex boundary conditions. The software provides a fully non-linear and path-dependent solution in the time domain. FLAC 3D uses the full equations of motion in the solution process. For static analysis, additional damping and mass scaling are introduced to facilitate the process of approaching static equilibrium. To obtain a dynamic solution, FLAC 3D uses the actual masses of the grid points and physically realistic damping. The nonlinearity and path dependence of the stress–strain response is determined by a constitutive law applied to each zone. For a complex constitutive law, modulus degradation and variation in damping due to strain variations are inherent to the constitutive law.

**Figure 10:**
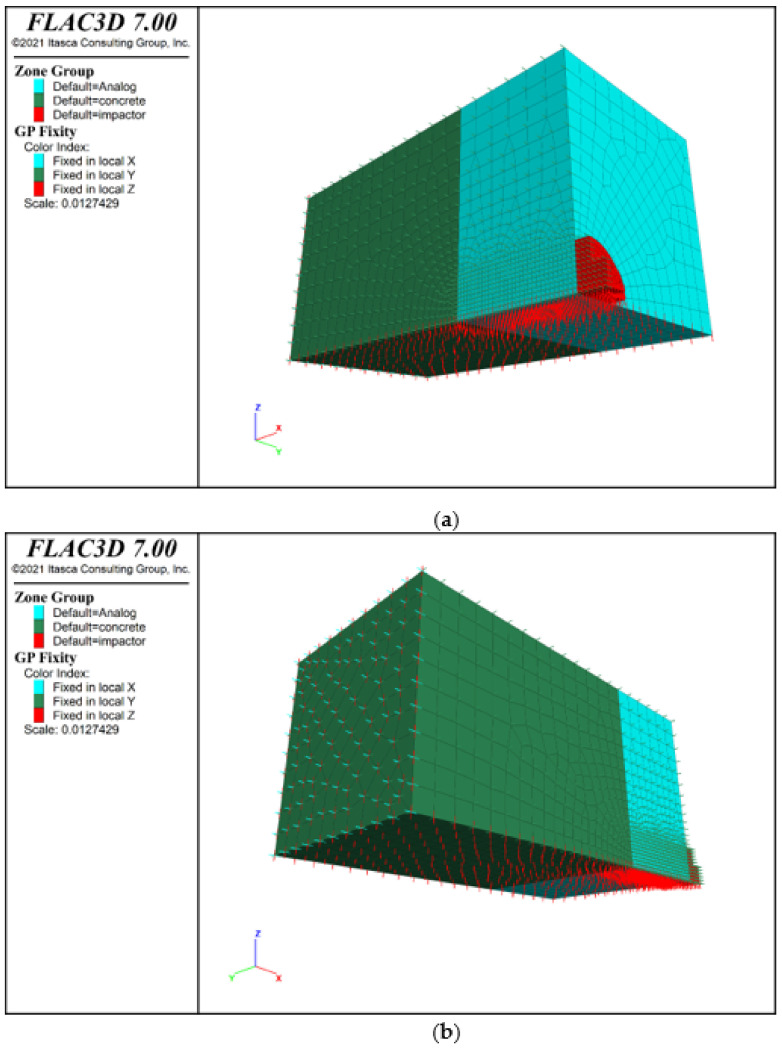
Numerical model of lander foot contact with analogue: (**a**) front view, (**b**) rear view.

The numerical model was implemented as a quadrant model (with two symmetry planes) in order to obtain reasonable computation times. Figure 10 illustrates an example of the geometry with boundary conditions for one of the LBT models [62]. The simulations were performed for a specified lander foot mass and assumed three predetermined lander foot velocities at the time of contact with the analogue surface. The calculations were conducted for foam-concrete analogues with thicknesses of 10 and 30 cm. As a result of numerical analysis, displacement, velocity, and acceleration diagrams were obtained for the whole time period of the contact process, i.e., from the moment of the first contact, stopping of the lander foot in the material, and its return path.

## 3. Results and Discussion

### 3.1. Laboratory Test Results of Foam Concrete

The laboratory examination of small samples provided a detailed determination of the physical and mechanical properties of the tested foam-concrete and concrete. A summary of the average results is presented in Table 2. The obtained results of the strength and deformation parameters unequivocally indicated a significantly higher stiffness of the concrete base in relation to the applied foam-concrete analogue, which supported the assumed model of the research material, which was to be characterized by a stiff base and a surface material in the zone of direct contact with the lander footing with significantly lower stiffness. The detailed results and test description can be found in the LOOP Report [63].

The laboratory results were used for numerical simulations of the contact phenomenon between the lander foot model and the Phobos analogue in the form of foam concrete slabs, the results of which are presented in Section 3.3.

### 3.2. Results of Two-Dimensional Vision System Measurements

**Figure 11:**
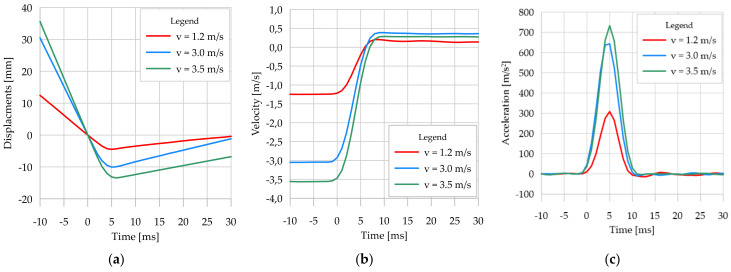
Results of lander foot contact measurements with a 30 cm thick analogue: (**a**) lander foot displacements; (**b**) lander foot velocities; (**c**) lander foot accelerations.

**Figure 12:**
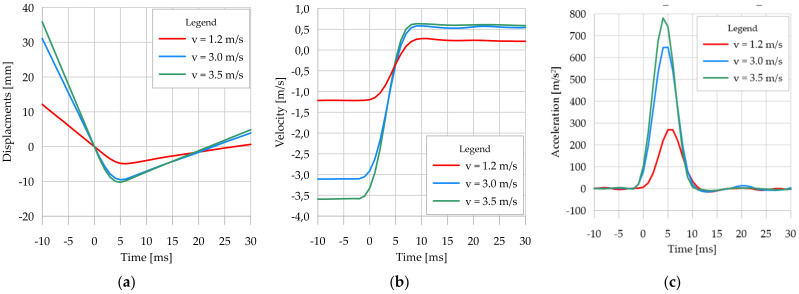
Results of lander foot contact measurements with a 10 cm thick analogue: (**a**) lander foot displacements; (**b**) lander foot velocities; (**c**) lander foot accelerations.

Table 3 presents the results of displacement and velocity measurements of the lander’s foot with the 2D vision system during its contact with the foam-concrete analogue. The results refer to the 30 cm and 10 cm thick analogues and the velocity of the lander’s foot in the moment of contact (contact velocity) at the following values, namely 1.2 m/s, 3.0 m/s, and 3.5 m/s, which are assumed to be descriptive for this type of test. It should be noted that the determined values of the lander foot velocity were specified time derivatives of displacements measured by the vision systems. The accelerations of the lander foot were calculated likewise. Due to the adopted method of contact initiation (reference measurement of the lander’s footprint), additional velocities of the lander’s foot in 1 and 2 ms before contact are given in Table 3. The results confirmed the accuracy of the method used to determine the beginning of contact, since in all cases, the lander’s foot speed decreased by more than 1.0%. At the same time, the lander’s velocity was close to constant and did not vary in time by more than 1.0% during the contact (Figure 11b and Figure 12b).

The contact duration, interpreted as the time interval between the beginning of contact and the deceleration of the lander foot in the analogue, was determined from the maximum displacement values of the lander foot. In principle, this duration was found to be independent of both the velocity at the time of contact and the thickness of the analogue itself. For all analyzed cases, the contact time was between 5 ms and 6 ms. However, it should be noted that due to the recording frequency of the measurements of 1000 fps (1 ms), the contact duration might have varied by less than 1 ms from that identified in the individual cases.

It should be highlighted that also after a rebound, the return of the lander foot at close to constant velocity was recorded. These velocities for the 30 cm thick analogue were lower than for the 10 cm thick analogue (Figure 11b and Figure 12b), indicating a higher kinetic energy dissipation of the 30 cm thick analogue. The reason for such behavior was the heavily porous structure of the foam-concrete, which was crushed upon displacement of the lander foot within it. The greater the thickness of the analogue, the greater the recorded crush zone, and thus the dissipation of impact energy, which translated into lower values of the lander foot velocity after the rebound. For comparative analysis, the time of 30 ms from the beginning of contact between the lander’s foot and the analogue was assumed. For a contact velocity of 1.2 m/s and an analogue thickness of 30 cm, a return velocity of 0.139 m/s was obtained, whereas for an analogue thickness of 10 cm, the return velocity of the lander foot was 0.211 m/s. Significantly increased differences in return velocity relative to analogue thickness were obtained for higher contact velocity values. For the contact velocity of 3.0 m/s and analogue thickness of 30 cm, the return velocity of the lander foot after the rebound was 0.361 m/s, and for the analogue of 10 cm thickness the return velocity of the lander foot after the rebound was 0.542 m/s. Similar dependencies were also obtained for the contact velocity of 3.5 m/s.

**Figure 13:**
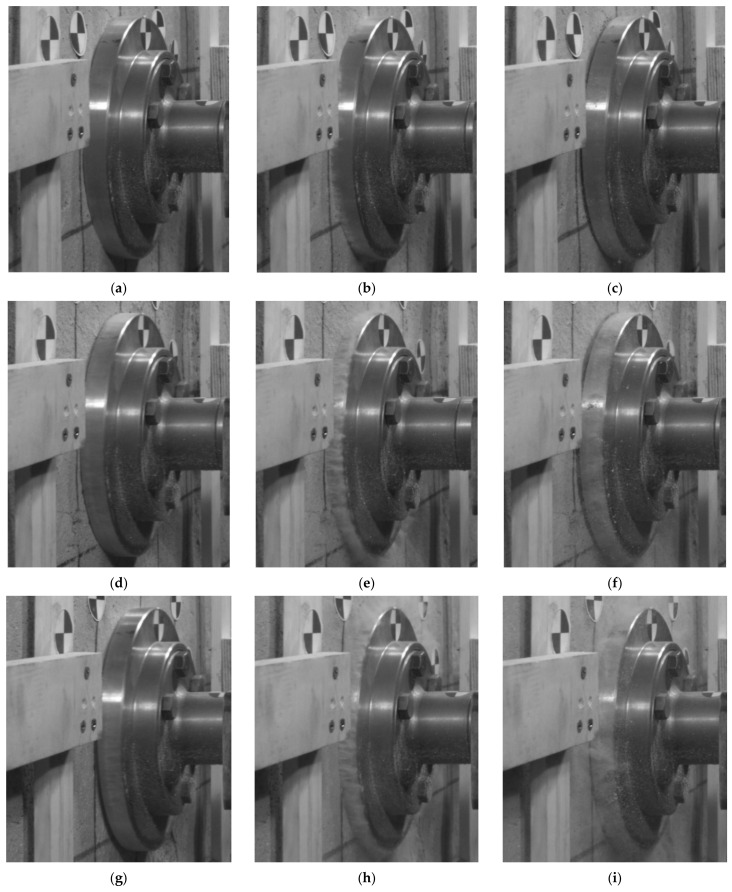
Lander foot positions in the 30 cm thick analogue tests: (**a**) at the contact point for a contact velocity of 1.2 m/s; (**b**) deceleration point of the lander foot for a contact velocity of 1.2 m/s; (**c**) the lander foot return point defined for 30 ms after contact for a contact velocity of 1.2 m/s; (**d**) at the contact point for a contact velocity of 3.0 m/s; (**e**) deceleration point of the lander foot for a contact velocity of 3.0 m/s; (**f**) the lander foot return point defined for 30 ms after contact for a contact velocity of 3.0 m/s; (**g**) at the contact point for a contact velocity of 3.5 m/s; (**h**) deceleration point of the lander foot for a contact velocity of 3.5 m/s; (**i**) the lander foot return point defined for 30 ms after contact for a contact velocity of 3.5 m/s.

**Figure 14:**
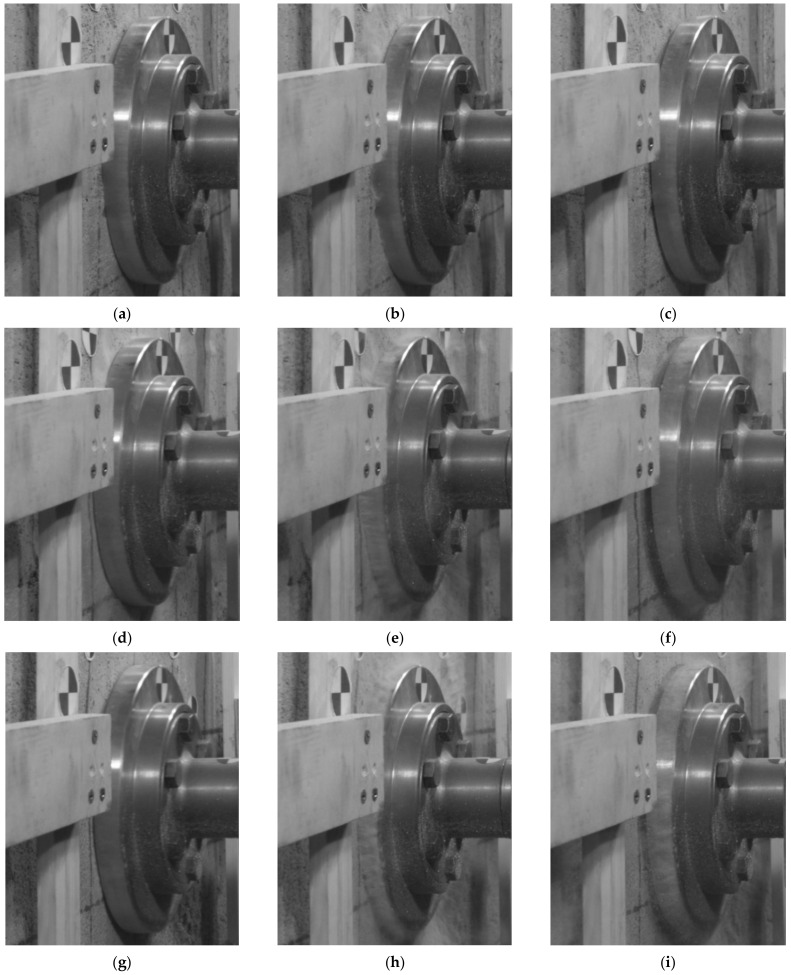
Lander foot positions in the 10 cm thick analogue tests: (**a**) at the contact point for a contact velocity of 1.2 m/s; (**b**) deceleration point of the lander foot for a contact velocity of 1.2 m/s; (**c**) the lander foot return point defined for 30 ms after contact for a contact velocity of 1.2 m/s; (**d**) at the contact point for a contact velocity of 3.0 m/s; (**e**) deceleration point of the lander foot for a contact velocity of 3.0 m/s; (**f**) the lander foot return point defined for 30 ms after contact for a contact velocity of 3.0 m/s; (**g**) at the contact point for a contact velocity of 3.5 m/s; (**h**) deceleration point of the lander foot for a contact velocity of 3.5 m/s; (**i**) the lander foot return point defined for 30 ms after contact for a contact velocity of 3.5 m/s.

The contact velocity had a substantial influence on the recorded analogue crushing, i.e., the higher the contact velocity of the lander foot, the greater the deformation recorded directly under the lander foot, due to the higher kinetic energy of the lander foot itself (Figure 11a and Figure 12a). However, it should be noted that with a larger analogue thickness, for the same value of contact velocity and thus kinetic energy, larger deformations were obtained directly under the lander foot. These differences for a contact velocity of 3.0 m/s were about 5%, and for a contact velocity of 3.5 m/s even 30%. Only for the low value of contact velocity of 1.2 m/s were the differences of recorded deformations in analogues of different thicknesses minor. This was explained by the low kinetic energy of the lander foot, which had a destructive effect only on the near-surface part of the analogues. In the case of a 10 cm thick analogue, for higher contact velocities, similar values of analogue deformation were obtained, resulting from the limitation of the crushing zone of the tested material related to its thickness. As a result of the dynamic impact of the lander foot, the analogue material was crushed, but due to its thickness and support on the rigid concrete material, the further increase in impact energy did not cause a proportional increase in the size of the deformation. The voids of the porous structure of the analogue were sealed, thus preventing any further increase in deformation. This behavior of the analogue material had a direct effect on the position of the lander foot after the rebound. For illustration purposes, in Figure 13 and Figure 14, the lander foot in three positions is shown for each test:

- direct contact with the analogue (value of 0 ms on the time axis of Figure 11a and Figure 12a);
- deceleration of the lander foot, thus obtaining the maximum displacement (values of 5–6 ms on the time axis of Figure 11a and Figure 12a);
- return of the lander foot determined in 30 ms after contact with the analogue (value of 30 ms on the time axis of Figure 11a and Figure 12a).

From the presented images, it was clear that for a 30 cm thick analogue for a contact velocity of 1.2 m/s and 3.0 m/s at 30 ms from the beginning of contact, the position of the lander’s foot did not differ much from its position at 0 ms. The additional edge on the surface of the analogue under the lander foot visible in Figure 13c,f in fact represents the resulting surface deformation of the analogue itself, not the displacement relative to the point of contact. On the other hand, for a contact velocity of 3.5 m/s, the lander foot was still inside the analogue in 30 ms after contact (Figure 13i). For a 10 cm thick analogue, only for a contact velocity of 1.2 m/s was a lander foot position at 30 ms after contact obtained such as at the point of contact (Figure 14c). This confirmed the previous statement that for small values of contact velocity, the kinetic energy obtained caused only near-surface deformation of the analogue and that it was hardly dependent on the thickness of the analogue itself. For contact velocities of 3.0 m/s and 3.5 m/s, the lander foot was outside the analogue in 30 ms after contact (Figure 14f,i), obtaining a similar position and return velocity (Figure 12b). The detailed 2D results concerning the whole scope of the project can be found in the LOOP Report [64].

**Figure 15:**
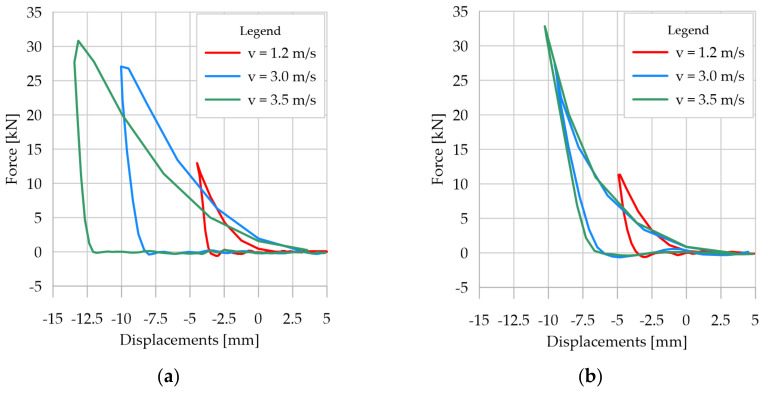
Relationship of the force acting on the lander foot as a function of its displacement relative to the surface of the analogue: (**a**) for a 30 cm thick analogue; (**b**) for a 10 cm thick analogue.

The analysis of the determined acceleration values (Figure 11c and Figure 12c) did not show any significant relationship of the analyzed parameter to the analogue thickness. Only for the contact velocity of 3.5 m/s was a 6.5% higher value of maximum foot acceleration obtained for the analogue with 10 cm thickness in comparison to 30 cm thickness. For the remaining contact velocities, similar maximum acceleration values were obtained for individual analogue thicknesses. Taking into account the principle that acceleration with which a point particle moves is proportional to the acting force, and the inverse of mass is the proportionality coefficient, the dependence of the force acting on the lander’s foot as a function of its displacement in relation to the analogue surface was determined. The results are presented in Figure 15, using the beginning of contact between the lander’s foot and the tested analogue as an origin point of the horizontal axis. The results clearly indicated different effects of the analogue on the lander foot, especially at higher contact velocities. For contact velocity of 1.2 m/s for different analogue thicknesses, the determined forces in the lander foot and the range of its displacement were similar. On the other hand, for contact velocities of 3.0 m/s and 3.5 m/s, in spite of obtaining similar force values, different dynamics of its increase should be emphasized. For the 10 cm thick analogue, the force increment in the lander foot was on average 25% higher for each 1.0 mm of foot penetration than for the 30 cm thick analogue. In the case of the 30 cm thick analogue, after deceleration, the force in the lander’s foot decreased rapidly, reaching 0 on the way back. In the case of the 10 cm thick analogue, the force decrease in the lander’s foot after deceleration was not so sudden, as evidenced by the slope of the plots in Figure 15b. In general, it can be concluded that at low contact velocities, and thus low kinetic energies, no significant differences in the behavior of the material directly under the lander foot were found for the analogue tested, and similar values of the forces in the lander foot were obtained. In the case of higher contact velocities, the behavior of analogues with different thicknesses was different, obtaining different values of analogue deformation, increase dynamics, and force decrease in the lander foot itself. The results may be of fundamental importance for engineers designing shock-absorbing systems for space landers.

### 3.3. Results of 3D Vision System Measurements

**Figure 16:**
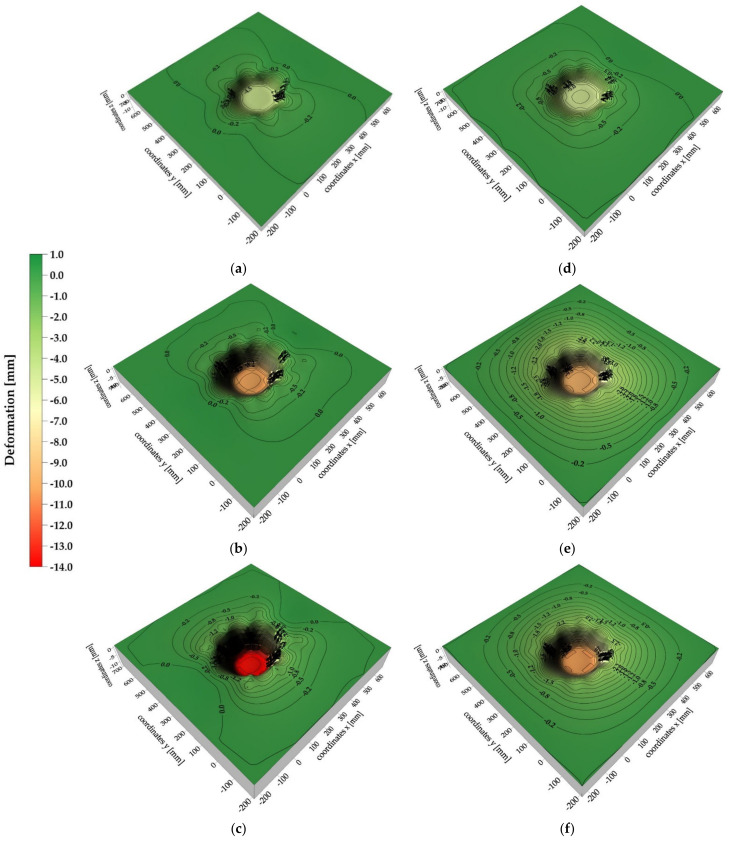
Maximum deformations of the tested analogues: (**a**) analogue thickness 30 cm, contact speed v = 1.2 m/s; (**b**) analogue thickness 30 cm, contact speed v = 3.0 m/s; (**c**) analogue thickness 30 cm, contact speed v = 3.5 m/s; (**d**) analogue thickness 10 cm, contact speed v = 1.2 m/s; (**e**) analogue thickness 10 cm, contact speed v = 3.0 m/s; (**f**) analogue thickness 10 cm, contact speed v = 3.5 m/s.

The analysis of the 2D vision system test results dealt with the behavior of the analogue only to a limited extent (analysis of the material directly under the lander foot). Therefore, in order to describe the behavior of the whole surface of the analogue, the analysis of the test results derived from 3D vision system was conducted. The analysis revealed that the deformation of the 30 cm thick analogue surface was asymmetrical (Figure 16a–c) with respect to one of the axes for all contact velocity cases. However, this could have been caused by the way the analogues with these thicknesses were mounted, which required additional fixings on the upper edges of the analogue.

For the analogues with thicknesses of 10 cm (Figure 16d–f), a more uniform and symetric distribution of deformation isolines was obtained. In general, it can be stated that in both cases, the increase of range of deformation zones with the increase of contact velocity was observed, as well as the occurrence of “stretching” of analogue material in the contact zone with the lander’s foot. However, it should be highlighted that, in reality, both “stretching” of the analogue material and its shearing at the contact edges with the lander’s foot occurred. However, due to the methodology used to create isolinear maps, the phenomenon of material shearing cannot be correctly mapped.

**Figure 17:**
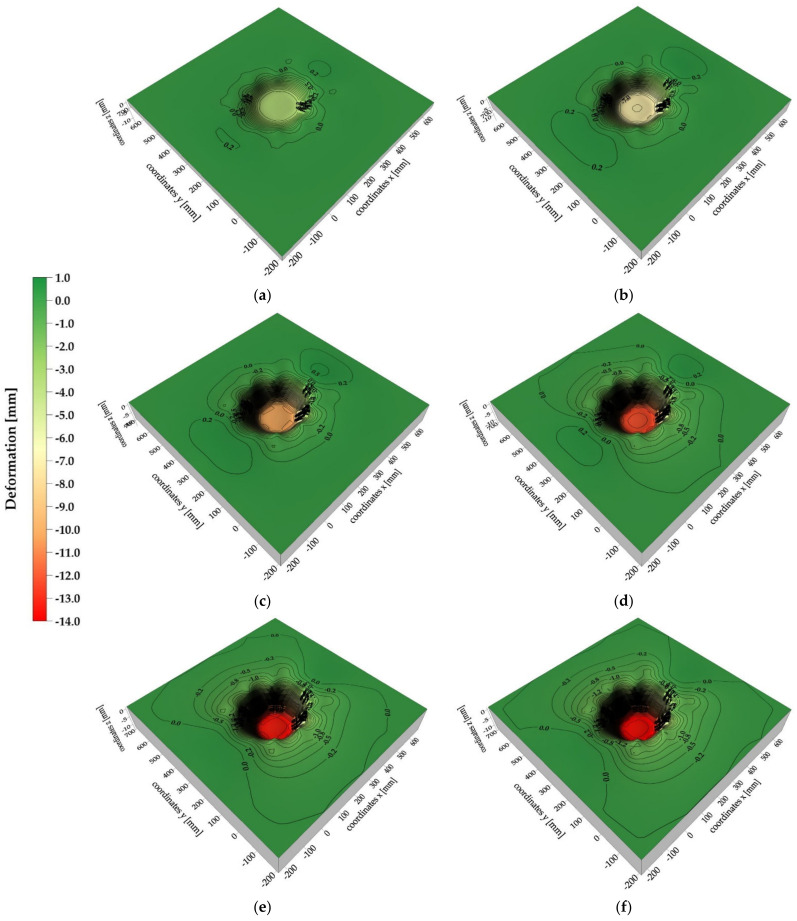
Surface deformation maps of a 30 cm thick analogue: (**a**) 1 ms after lander foot contact; (**b**) 2 ms after lander foot contact; (**c**) 3 ms after lander foot contact; (**d**) 4 ms after lander foot contact; (**e**) 5 ms after lander foot contact; (**f**) 6 ms after lander foot contact.

**Figure 18:**
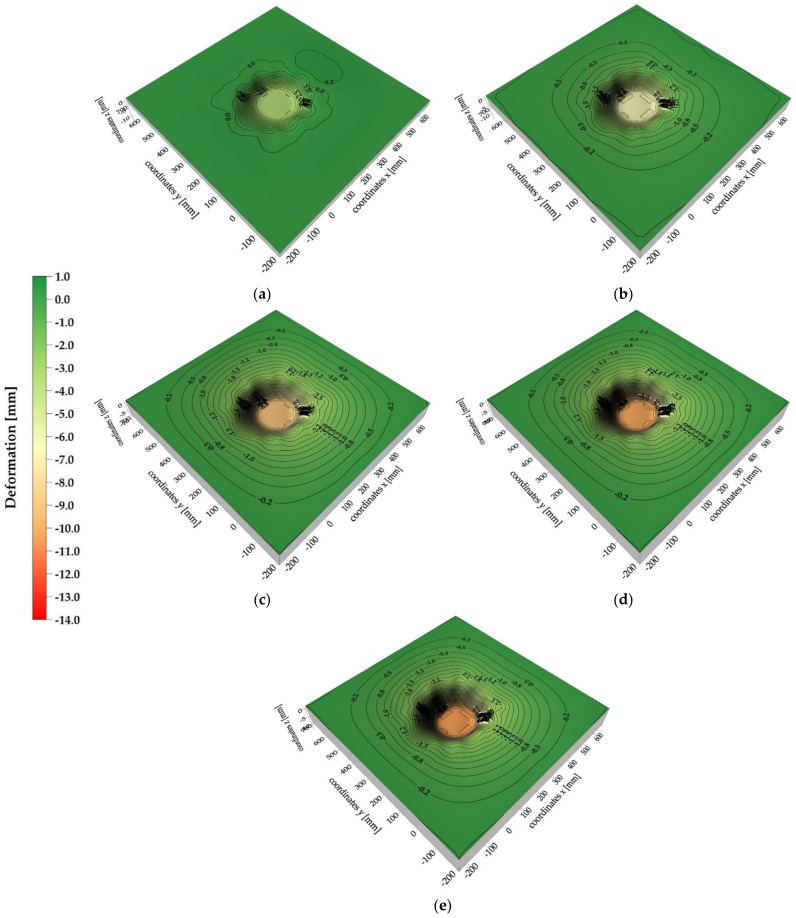
Surface deformation maps of a 10 cm thick analogue: (**a**) 1 ms after lander foot contact; (**b**) 2 ms after lander foot contact; (**c**) 3 ms after lander foot contact; (**d**) 4 ms after lander foot contact; (**e**) 5 ms after lander foot contact.

For material thicknesses of 30 cm (Figure 17) and 10 cm (Figure 18), surface deformation maps of the analogues for contact velocities equal to 3.5 m/s at time intervals of 1 ms are presented. Both negative and positive deformations were obtained for the 30 cm thick analogue from 1 ms to 4 ms after contact. The positive isolines may have originated from local phenomena related to the surface wave, which did not propagate symmetrically over the surface due to the way the analogue was mounted. In the time of 5–6 ms after contact, only negative isolines were obtained, indicating the indentation of almost the whole surface of the analogue. For the 10 cm thick analogue, only in 1 ms after contact were positive deformation values obtained. In the remaining cases, an approximately symmetrical increase in the analogue surface indentation was recorded. It should be underlined that all obtained analogue surface deformation maps were created using the interpolation method and may slightly differ from the real deformation patterns. Nevertheless, the presented tool provided a general picture of the analogue surface behavior as a result of a contact with the lander foot. The accuracy of the deformation maps strictly depends on the number of measurement points.

The detailed 3D results concerning the whole scope of the project can be found in the LOOP Report [64].

### 3.4. Numerical Simulation Results

**Figure 19:**
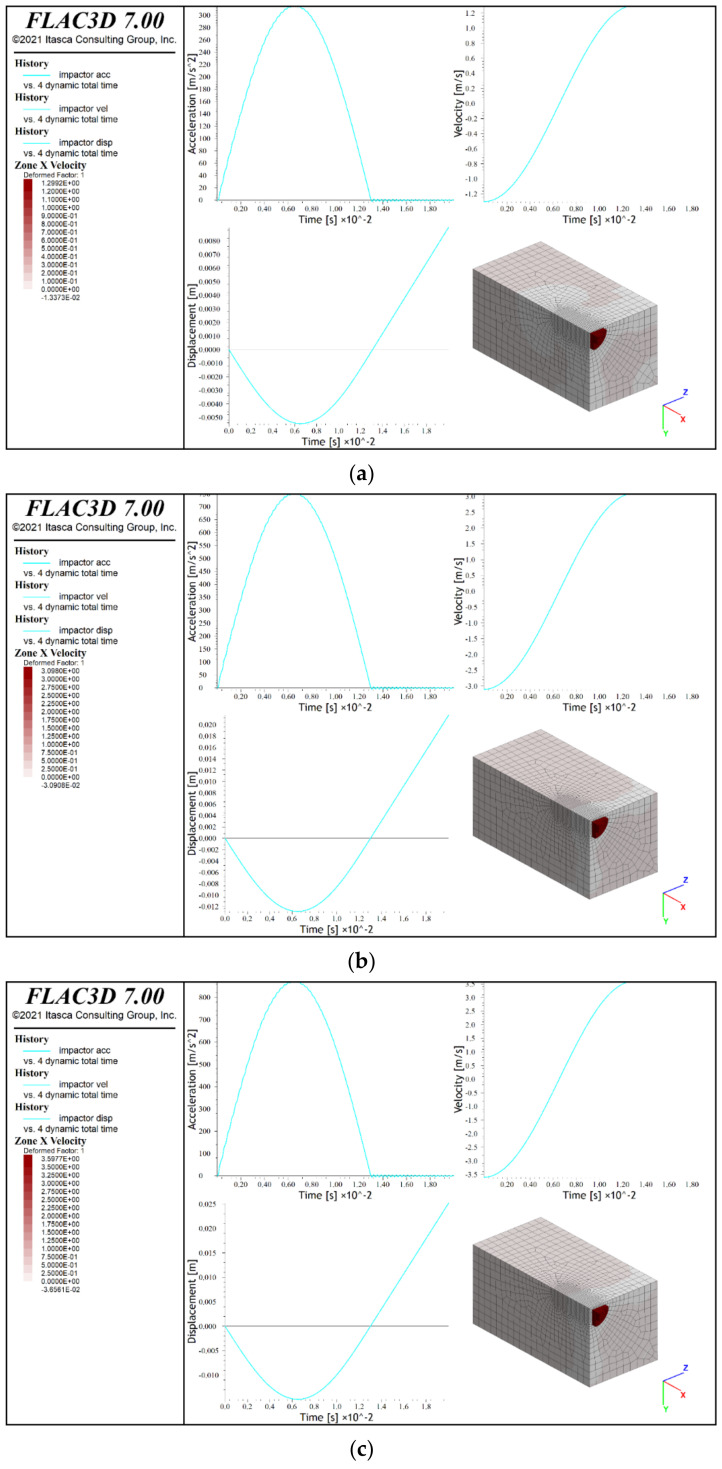
Results of numerical simulations of lander foot contact with a 30 cm thick analogue: (**a**) contact velocity v = 1.2 m/s; (**b**) contact velocity v = 3.0 m/s; (**c**) contact velocity 3.5 m/s.

**Figure 20:**
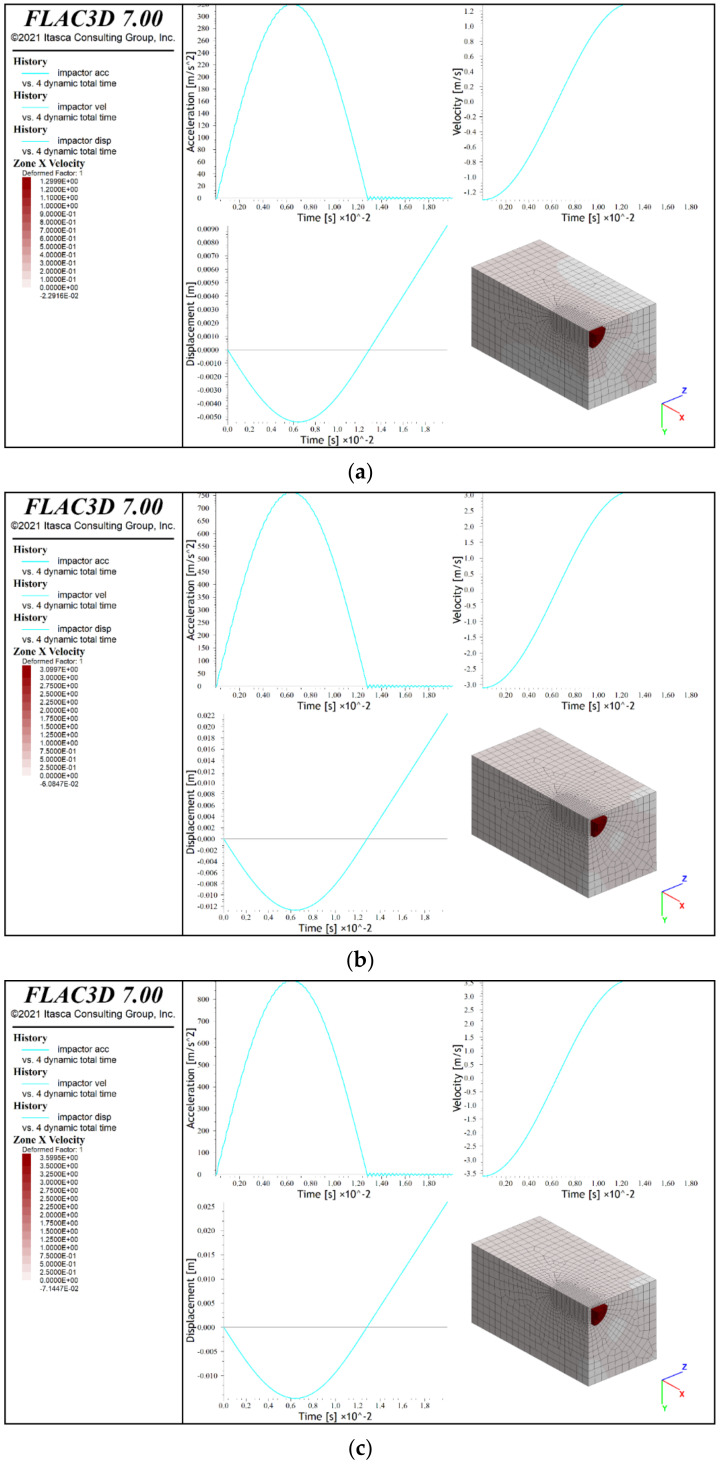
Results of numerical simulations of lander foot contact with a 10 cm thick analogue: (**a**) contact velocity v = 1.2 m/s; (**b**) contact velocity v = 3.0 m/s; (**c**) contact velocity 3.5 m/s.

The numerical calculations showed the maximum displacements of the lander foot in the analyzed tests and the corresponding maximum accelerations. The obtained results are presented graphically in Figure 19 and Figure 20, where in addition to the diagrams of individual relationships, the deformation distributions of the analogues are given. Table 4 presents a comparison of the obtained test results and numerical simulations.

The analysis of the results indicated that the phenomena occurring during the dynamic contact of the lander’s foot with the surface of the tested analogue could be correctly reproduced only to a limited extent. The best agreement of the results was obtained for the maximum values of the lander foot acceleration. However, it should be noted that the maximum differences in the values determined during the tests and on the basis of numerical simulations reached up to 20%. In the case of the remaining parameters, including primarily the lander foot displacement, the results were characterized by an even greater discrepancy. It might have been caused by simplifications of the numerical model, which did not take into account, such as the following:

- the layered arrangement of the concrete slabs that underlie the analogues and the contact conditions between the slabs;
- friction and other movement resistances of the lander foot;
- the additional fixing of 30 cm thick analogues;
- shearing of the analogue material when in direct contact with the lander foot;
- the lack of actual parallelism of the surface of the analogue with the surface of the lander foot;
- misalignment of the actual perpendicularity of the camera’s optical axis to the plane of the runner element in the vision-based calculation of the marker displacement on the runner element.

Therefore, it is necessary to carry out further calibration of the developed numerical models in order to achieve greater consistency of simulation results in relation to the test results. However, considering the imperfections of the developed numerical models, a certain degree of convergence regarding the deformation of the analogue surface in numerical simulations could be observed. For a 30 cm thick analogue, radial directions of surface deformation were found for all contact velocities (bright zones in y and z directions of the local coordinate system). On the other hand, for 10 cm thick analogues, centric deformation systems prevailed. Similar relationships were observed in the experiments, which confirmed the correct direction of numerical modelling to describe the analyzed phenomenon.

However, it should be emphasized that the adopted FDM computational model, similarly to the case of the developed interpolation maps of the analogue surface deformation, did not accurately describe the shear phenomenon at the contact edges of the lander foot with the analogue. This is due to the continuity of the computational model and the FDM method itself. In this method, the failure zones can be defined as material plasticization zones, which, however, do not show the discontinuity of the model material itself. The solution to this problem may be the use of other computational tools. In further work it is assumed to use the distinct element method (DEM), in particular Particulate Flow Code (PFC) software, which provides a few constitutive models of soils. At this stage we can only presume which model would be the most suitable for analogue. For this reason, the most representative model for analogue (loose, non-cohesive material) will be chosen based on laboratory tests results. It is also worth mentioning that both FLAC and PFC have the special modules called “user defined constitutive models”, which means one may introduce one’s own models (most appropriate to describe the mechanical behavior of footpad or analogue) into the commercial code. Nevertheless, it should be highlighted that the crux of the issue of modeling the contact phenomena between the lander’s foot and the surface of the analogue using the PFC method is the correct determination of the contact conditions between individual particles.

## 4. Conclusions

The prediction of the material properties and their behavior during the contact with another object (e.g., a lander), as well as the lander itself, is an extremely significant element determining the success of projects related to the exploration of celestial bodies (planets, the Moon, moons of planets, etc.). The success of individual space missions will often be dependent on properly designed shock-absorbing systems, which enable landers to safely land on celestial bodies with different gravity levels, shapes, and surface structures. Therefore, the possibility of simulating the phenomenon of contact between the lander’s foot and the space analogue under laboratory conditions increases the chances of success for future space missions.

Performed simulation investigations required the creation of a test stand, which would provide kinetic energy mapping with which a single lander’s foot in a planned landing mission on one of Mars’ moons, Phobos, could impact the terrain of different stiffness and structure. Due to the assumed perpendicular direction of the lander’s foot in relation to gravity, the test can be carried out only for compact materials.

Given the short contact duration of the lander foot with the analogues, an important challenge was to use a suitable measurement system that would give sufficient quality of results relating to both the lander foot itself and the behavior of the analogue under investigation. The 2D and 3D vision systems adopted for this purpose satisfactorily met these expectations. The 2D vision system measured the motion parameters of the lander foot, which allowed us to describe its behavior before, during, and after the contact with a given analogue, whereas the 3D vision system, by determining three-dimensional displacements in the set measurement points, allowed us to describe the behavior of the analogue surface during tests in specific time intervals. Analysis of the measurement results indicated that the advantage of the adopted measurement solution was the possibility of simultaneous observation of individual elements of the lander’s foot-analogue system.

The results showed that at low contact velocities, and thus low kinetic energies, no significant differences in behavior of the material directly under the lander foot were observed, and similar values of forces in the lander foot were obtained. For higher contact velocities, the behavior of analogues with varying thicknesses was different, resulting in different values of analogue deformation and dynamics of increments and decrements of forces in the lander foot itself.

Despite testing the same material, the experiments showed different behavior depending on the thickness of the material. This is an important point for engineers, who should take this fact into account in the process of designing the lander itself. Minimizing the influence of the analogue thickness on the contact between the lander’s foot and the analogue, in light of the conducted tests, could be achieved by reducing the kinetic energy, i.e., decreasing the lander’s mass or its velocity during landing.

The experimental tests were complemented by their numerical simulations, which, after appropriate calibration, would allow the simulation of materials with different thicknesses and contact velocities. However, it should be noted that the developed numerical models require further specification with respect to the experimental results in order to fully support the lander foot design process and more closely represent the real phenomenon. Using other computational methods also seems appropriate. In future work, it is planned to use the PFC, which may give more reliable results. However, it should be kept in mind that the essence of the problem of modeling the contact phenomena between the lander’s foot and the surface of the analogue using the PFC method is the correct determination of the contact conditions between individual particles.

Furthermore, it is desirable to conduct tests on other materials, which will differ significantly in their structure and physical and mechanical parameters. Due to the lack of information on the surface of Phobos, it is possible to analyze materials similar to those for which properties were determined on the basis of previous planetoid missions. However, one should keep in mind the limitation that only cohesive materials can be tested on the designed test stand.

A separate problem, which should be analyzed in further works, is mapping the contact of the lander’s foot with the surface of the analogue at different angles. Considering the expected roughness of the Phobos surface, it is suggested to perform additional tests, which will allow the contact phenomenon to be described as for the surface perpendicular to the direction of the lander’s foot motion. The test stand and the developed measuring system allow one to perform such tests, and the analyses of the test results can be a significant contribution to the design of shock absorbing systems for landers’ feet.

## Figures and Tables

**Table 1:** Specification of the vision systems and additional equipment.

2D Vision System	3D Vision System
One high-speed camera (Phantom VEO 340L) equipped with: Lens: Carl Zeiss Focal length 25 mm; Aperture: F/8	Two high-speed cameras (Phantom v9.1) equipped with: Lens: Carl Zeiss Focal length 50 mm Aperture: F/8
Settings: Frame rate: 1000 fps Image resolution: 2048 × 1536 Exposure: from 400 µs Distance between camera and an analogue: 898 mm	Settings: Frame rate: 1000 fps Image resolution: 1632 × 800 Exposure: from 850 µs Distance between left camera and an analogue: ~1.5 m
A lighting system: Two halogen lamps (2 × 500 W)	
Image-based processing software: Tema-Automotive [60]	

**Table 2:** Properties of tested materials.

Properties of Materials	Foam Concrete	Concrete
Bulk density (g/cm^3^)	0.80	2.24
Uniaxial compressive strength (MPa)	6.30	49.53
Splitting tensile strength (MPa)	1.25	3.23
Modulus of elasticity (GPa)	6.21	34.12

**Table 3:** Results of 2D vision system investigation.

Test No.	I	II	III	IV	V	VI
Thickness of analogue/concrete (cm)	30/70	30/70	30/70	10/90	10/90	10/90
Contact duration (ms)	5	5	6	6	5	5
Velocity at the time of contact (m/s)	−1.216	−2.924	−3.466	−1.196	−2.910	−3.326
Velocity relative to the 1 ms before beginning of contact (m/s)	−1.239	−3.028	−3.537	−1.211	−3.050	−3.520
Velocity relative to the 2 ms before beginning of contact (m/s)	−1.244	−3.044	−3.556	−1.213	−3.071	−3.541
Maximum displacement of the lander foot from the moment of contact with analogue (mm)	4.485	10.035	13.439	4.918	9.547	10.259

**Table 4:** Comparison of the obtained test results and numerical simulations.

Test No.	I	II	III	IV	V	VI
Thickness of analogue/concrete (cm)	30/70	30/70	30/70	10/90	10/90	10/90
Velocity during contact (experiments) (m/s)	−1.216	−2.924	−3.466	−1.196	−2.911	−3.326
Velocity during contact (numerical simulations) (m/s)	−1.2	−3.0	−3.5	−1.2	−3.0	−3.5
Contact duration (experiments) (ms)	5	5	6	6	5	5
Contact duration (numerical simulations) (ms)	6.4	6.4	6.5	6.4	6.4	6.4
Maximum lander foot displacement since contact with analogue (experiments) (mm)	4.485	10.035	13.439	4.918	9.547	10.259
Maximum lander foot displacement since contact with analogue (numerical simulations) (mm)	5.4	12.5	14.9	5.3	12.5	14.8
Maximum lander foot acceleration since contact with analogue (experiments) (m/s^2^)	308.341	643.614	733.067	269.583	646.673	780.951
Maximum lander foot acceleration since contact with analogue (numerical simulations) (m/s^2^)	320	750	880	320	750	880

## Data Availability

Data sharing not applicable due to external funding.
